# Availability of stock epinephrine in schools is needed to address health disparities in anaphylaxis

**DOI:** 10.1016/j.jacig.2022.06.003

**Published:** 2022-08-12

**Authors:** Mary K. Vetter, Ashley C. Newell, Margaret P. Huntwork, John C. Carlson

**Affiliations:** a Tulane University School of Medicine, New Orleans, La; b Ochsner Medical Center, Jefferson, La

**Keywords:** Anaphylaxis, stock epinephrine, schools, advocacy, health disparities

## Abstract

Significant health disparities exist in the diagnosis, prevalence, and management of anaphylaxis. This case acted as a community-level sentinel event for advocacy efforts to place stock epinephrine into schools throughout the greater New Orleans area.

In 2011, an adolescent Black high school student experienced a syncopal event in her classroom as she was returning from lunch with classmates. Our mobile clinic team was parked outside the school to increase access to medical care while clinics were being rebuilt from the flooding that followed Hurricane Katrina, and it was summoned to help. On examination, the girl was supine on the floor and unarousable. She had a normal respiratory rate with diffuse inspiratory and expiratory wheezes. Her skin was without urticaria, angioedema, or flushing. There was no evidence of vomiting. According to her friends, she was known to have a fish allergy with previous episodes of anaphylaxis, but there were no known exposures during the lunch period.

The student did not have an epinephrine autoinjector (EAI) at the school. The school did not carry unassigned stock epinephrine (ie, EAIs prescribed to the school to be used on any student in need). The school nurse noted that she could not legally provide another child’s EAI to use on this child. An EAI was brought from the mobile clinic and deployed through the student’s pants into the anterolateral thigh. Ten minutes later an ambulance arrived and took the child, still unconscious, to the emergency department. Because our team had not previously cared for the patient and no consent forms had been completed by the patient’s family, Federal Educational Rights and Privacy Act regulations prevented the school from providing our team with additional information on the outcomes of this case.

Louisiana law allows a physician to prescribe unassigned stock EAIs to schools, provided that the school has adopted a policy for EAI use and school personnel have been appropriately trained. Since this case, our mobile clinic team has worked with public schools throughout the greater New Orleans area to ensure adoption of appropriate policies, completion of training, and prescription of EAIs that have been used to treat children with episodes of anaphylaxis in schools. School administrators are often understandably nervous about the risks associated with having stock EAIs; the law allows for the presence of EAIs but does not require it, and there are no specific legal “Good Samaritan” protections in place for staff who use the EAIs in good faith. We acknowledge this concern in our training. In advocacy training, physicians are told that patient stories are among the most powerful tools for creating change. Describing this case, including the worry that we had for the life of this patient, has been useful in demonstrating how important stock EAIs are to addressing the health disparities of underserved communities that these schools serve despite the lack of legislation requiring EAIs. Ten years after this case, our team has trained staff, edited policies, and prescribed epinephrine to 55 of the 90 public schools in New Orleans.^1^

## Discussion

The diagnosis of anaphylaxis is made when multiple organ systems are affected after exposure to a probable allergic trigger; 1 of the organ systems must be the mucocutaneous system if there was no likely trigger present. In the case of a patient with a history of anaphylaxis, a presumptive diagnosis can be made when only the cardiovascular system is involved. Epinephrine is considered the first-line therapy for cases of anaphylaxis, with EAIs as the medication of choice for cases in the community. Time to treatment with epinephrine is a crucial factor in survival during anaphylaxis.^2^ In this case of anaphylactic shock, epinephrine helps support cardiovascular function as the most immediate need, and it reduces vascular leak, which reduces ongoing loss of intravascular volume. Although rare, most fatalities occur outside medical facilities and are associated with delayed epinephrine administration.

In the case of this Black student, it is important to consider racial disparities as a factor in the outcome of this event. Racial disparities can be seen in children with allergic diseases as early as at age 2 years even when adjusted for other factors such as socioeconomic status.^3^ In an analysis of fatal anaphylaxis cases in the United States, a significant association with Black race was found regarding events related to most categories of allergens.^4^ Increased cases of drug-induced anaphylaxis may reflect higher rates of comorbidities, more use of and exposure to medications, and less access to care.^4^ Black children are not the only racial group affected by these disparities. The proportion of non-Hispanic Black and Hispanic infants and toddlers hospitalized for anaphylaxis is rising; worsening outcomes in cases of anaphylaxis have also been seen in these racial/ethnic groups.^5^

Increasing access to EAIs is key to addressing poor outcomes and reducing health disparities in anaphylaxis. In schools in which unassigned stock EAIs are available, EAIs from this stock are used more often than those from a student’s assigned supply.^6^ Use of unassigned EAIs has been shown to be cost-effective and improve health outcomes.^6^^,^^7^ Unfortunately, the number of EAIs in schools is inversely proportional to the number of students who qualify for free or reduced-price lunch.^8^ It is also important to note that Black and Asian children are less likely to have a formal allergy diagnosis, despite the increased prevalence of allergy in these groups.^9^ These children may benefit most from unassigned stock EAIs, as they are less likely to have prescribed EAIs without this formal diagnosis. When campaigning for universal stocking in schools, it may be particularly effective to target districts with the most at-risk children.

## Conclusion

Epinephrine is the first-line treatment for anaphylaxis, with early administration being an important goal. Increasing the availability of unassigned stock EAIs in schools is an important strategy to improve access for all children, and broad implementation may help reduce the striking health disparities seen in anaphylaxis outcomes. This case report is an example of how physicians can use case examples in their advocacy efforts to influence policies affecting communities.
